# Anti-malarial activity of *Holarrhena antidysenterica *and *Viola canescens*, plants traditionally used against malaria in the Garhwal region of north-west Himalaya

**DOI:** 10.1186/1475-2875-10-20

**Published:** 2011-02-02

**Authors:** Gaurav Verma, Virendra K Dua, Dau Dayal Agarwal, Pravin Kumar Atul

**Affiliations:** 1 National Institute of Malaria Research, Field Unit Hardwar-249403, India; 2 National Institute of Malaria Research, Sector-8, Dwarka, New Delhi-110077, India; 3 School of Studies in Chemistry, Jiwaji University, Gwalior, 474 011, India

## Abstract

**Background:**

The increasing number of multidrug-resistant *Plasmodium *strains warrants exploration of new anti-malarials. Medicinal plant research has become more important, particularly after the development of Chinese anti-malarial drug artemisnin from *Artemisia annua*. The present study shows evaluation of anti-malarial effects of two plants commonly used against malaria in the Garhwal region of north-west Himalaya, in order to discover the herbal-based medicine.

**Methods:**

*In vitro *anti-plasmodial sensitivity of plant extracts was assessed using schizont maturation and parasite lactate dehydrogenase (pLDH) assay. Cytotoxic activities of the examined extracts were determined on L-6 cells of rat skeletal muscle myoblast. The 4-day test for anti-malarial activity against a chloroquine sensitive *Plasmodium berghei *NK65 strain in Swiss albino mice was used for monitoring *in vivo *activity of plant extracts.

**Results:**

Chloroform extract of *H. antidysenterica *(HA-2) and petroleum ether extract of *V. canescens *(VC-1) plants significantly reduced parasitaemia in *P. berghei *infected mice. The extract HA-2 showed *in vitro *anti-plasmodial activity with its IC_50 _value 5.5 μg/ml using pLDH assay and ED_50 _value 18.29 mg/kg in *P. berghei *infected Swiss albino mice. Similarly petroleum ether extract of *V. canescens *(VC-1) showed *in vitro *anti-plasmodial activity with its IC_50 _value 2.76 μg/ml using pLDH assay and ED_50 _15.8 mg/kg in *P. berghei *infected mice. The extracts coded as HA-2 at 30 mg/kg and VC-1 at 20 mg/kg exhibited parasite inhibition in mice: 73.2% and 63.0% respectively. Of these two plant extracts, petroleum ether extract of *V. canescens *was found slightly cytotoxic.

**Conclusion:**

The present investigation reflects the use of these traditional medicinal plants against malaria and these plants may work as potential source in the development of variety of herbal formulations for the treatment of malaria.

## Background

Despite the incessant global efforts to fight parasitic infections and the attempts to eliminate the causative organisms, malaria still remains as one of the greatest human killers, causing almost 1 million deaths per year (mainly small children in Africa) and 300-400 million infections annually [1]. In the South East Asian Region of WHO, people living in eleven countries are exposed to the risk of malaria and most of whom (more than 78%) live in India [2].

The emergence of drug resistance is reducing the therapeutic arsenal for the treatment of malaria at a rate that is barely balanced by the development of novel effective drugs. In this regard medicinal plant research has become more important particularly after the development of Chinese anti-malarial drug artemisinin, isolated from *Artemisia annua*, a drug from traditional medicinal plants [3,4]. Natural plant products are main sources of biologically active compounds and have potential for the development of novel anti-malarial drugs. More recently a number of studies have been undertaken to evaluate the inhibitory effects of various plant extracts on *Plasmodium falciparum *[5,6] and *Plasmodium berghei *[7,8].

Two plants *Holarrhena antidysenterica *(Apocynaceae) and *Viola canescens *(Violaceae) commonly used as traditional medicine in the Garhwal region of north-west Himalaya for the treatment of protozoan infections and fever including malaria were studied. The stem bark of the *H. antidysenterica *plant, commercially known as kurchi and kutaz in the Indian subcontinent has been investigated due to its traditional use in the treatment of amoebic dysentery, diarrhoea, asthma, bronchopneumonia [9,10]. In addition the plant has been reported to possess anti-helminthic, appetizing, anti-diarrhoeal, astringent properties [11], used as an immunomodulating agent [12], larval growth inhibitor [13] and against vaginitis [14]. Gaur [15] reported that the bark of the *H. antidysenterica *is used against malaria in the Garhwal region of north-west Himalaya. The other plant, *V. canescens *commonly known as Gull-e-Banafsha and Himalayan White Violet in Indian herbal market is a nearly prostrate herb found at altitudes of 1,500-2,400 m in the Himalayan region. The plant is extensively used against cough, fever and malaria [15].

These plants were tested for their *in vitro *and *in vivo *anti-malarial activity. In order to determine the selective indexes, cytotoxic activities of these plants were also studied.

## Methods

### Collection of plants

Based on ethnophamocological and ethnobotanical literature [15] plants were collected during flowering season of year 2008 from the Garhwal region (*H. antidysenterica *from Cheela range and *V. canescens *from Chamba) and identified by Botanical Survey of India, Dehradun, India. Voucher specimens of the plants were stored in the Institute herbarium (voucher specimen numbers NIMRHAR-101-VC for *V. canescens *and NIMRHAR-101-HA) for future reference.

### Plant extract preparation

The collected plants were washed with distilled water and dried on absorbing paper at an ambient temperature (25-30°C) in open air under shade for 5-10 days. The dry plants samples were ground to powder and stored at ambient temperature until use. For each plant, four types of extracts were prepared (petroleum ether, chloroform, methanol and water) in accordance with the increasing polarity of these solvents. The extracts were prepared by maceration of the plant material in the solvent at room temperature (50 g of powder in 150 ml of solvent) for three successive periods of three days, with a change of solvent every two days and then concentrated under reduced pressure. All extracts were tested for their residual solvents by GCMS analysis and solvent free extracts were screened for anti-malarial activity (*in vitro *and *in vivo*).

### *In vitro *anti-plasmodial sensitivity against *P. falciparum *isolates

*In vitro *anti-plasmodial sensitivity of plant extracts was assessed at National Institute of Malaria Research, Dwarka, New Delhi, India using the procedure of Trager and Jensen [16]. These extracts were also sent to WHO, Switzerland for screening of *in vitro *anti-plasmodial activity using the parasite lactate dehydrogenase (pLDH) assay developed earlier [17]. *Plasmodium falciparum *chloroquine sensitive strain FSG derived from an Indian patient of Shahjahanpur (UP) was used for the study using *in vitro *candle-jar method. Culture was maintained in A +ve erythrocytes using RPMI 1640 medium supplemented with AB Rh +ve human serum (10%), sodium bicarbonate (0.2%), HEPES buffer (25 mM) and gentamycin (50 μg ml^-1^). The culture was treated with selected concentrations of extracts. Blood smears were prepared and stained with Giemsa strain after 72 hrs of incubation % maturation of schizonts against control was recorded. Chloroquine was taken as positive control. The inhibitory concentration value, kills 50% of the parasites (IC_50_) was considered for anti-plasmodial activity. For the parasite lactate dehydrogenase (pLDH) assay, chloroquine sensitive GHA strain derived from a Ghanaian patient was used and maintained in RPMI 1640 medium supplemented with 0.37 mM hypoxanthine, 25 mM HEPES, 25 mM NaHCO_3 _and 10% A+ve human serum together with 2-4% washed human O +ve erythrocytes. All cultures and assays were conducted at 37 ± 1°C and an atmosphere of 4% carbon dioxide, 3% oxygen and 93% nitrogen. Assays were performed in sterile 384- well microtiter plates, each well containing 2 μl of compound solution (selected concentration) with 38 μl of the parasite inoculums (1% parasitaemia, 2% haematocrit). Parasite growth was compared to control wells (100% parasite growth). Plates were deep-frozen at -20°C, after 72 h of incubation at 37 ± 1°C. After thawing 5 μl from each well was transferred into another plate together with 25 μl of Malstat ™ reagent and 5 μl of a 1/1 mixture of PES (phenazine ethosulfate, 2 mg/ml) and NBT (Nitro Blue Tetrazolium Grade III, 0.1 mg/ml). The plates were then kept out of light for 2 h and the change in colour was measured with a spectrophotometer at 655 nm. The results were expressed as percent reduction in parasitaemia compared to control wells. Chloroquine was taken as positive control.

### *In vivo *anti-malarial sensitivity against *P. berghei*

The test described by Peters [18] for anti-malarial activity against a chloroquine sensitive *P. berghei *NK65 strain in Swiss albino mice was used for monitoring *in vivo *anti-malarial activity of extracts coded as VC-1 and HA-2. The protocol was approved by the animal ethical committee of the National Institute of Malaria Research, Delhi, India and the experiments were performed under the supervision of a veterinary doctor. The infected erythrocytes were injected intraperitonially (1 × 10^7 ^parasite per mouse) on day 0. The drug was administered intraperitonially for four consecutive days from day 0 to day 3 (10-30 mg/kg) in the experimental group. The control group was given the solvent (DMSO) in equal volume for the same duration. On day 4, tail blood smears were prepared, stained and percent parasitaemia was recorded. The suppression of parasitaemia in experimental group as compared to control group was calculated. Chloroquine was taken as positive control. The suppression of parasitaemia in relation to the control was assessed using the formula by Obih *et al *[19].

### Cytotoxicity on rat skeletal muscle myoblasts (L-6 Cells)

The cytotoxicity of the examined extracts was determined using method reported earlier [20,21]. Rat skeletal muscle myoblasts, cell line L-6, were seeded in 96-well Costar microtiter plates at 2 × 103/cells/100 ml, 50 ml per well in MEM supplemented with 10% heat inactivated FBS. A three-fold serial dilution ranging from 90 to 0.13 mg/ml of compounds in test medium was added. Plates with a final volume of 100 ml per well were incubated at 37 1C for 72 h in a humidified incubator containing 5% CO_2_. Resazurin was added as viability indicator. After an additional 2 h of incubation, the plate was measured with a fluorescence scanner using an excitation wavelength of 536 nm and an emission wavelength of 588 nm (SpectraMax GeminiXS, Molecular Devices).

### Data analysis

Collected data from *in vivo *anti-malarial activity were subjected to analysis of Variance (ANOVA) by Scheffe's procedure using the software StatPlus 2009 Professional.

## Results

### *In vitro *anti-plasmodial actions of the plant extracts against *P. falciparum *isolates

The anti-plasmodial activities of extracts coded as HA-1, HA-2, HA-3 and HA-4 from *H. antidysenterica *and VC-1, VC-2, VC-3 and VC-4 from *V. canescens*, are given in Table 1. The extracts HA-2 from *H. antidysenterica *and VC-1 from *V. canescens *possessed better anti-malarial activity with IC_50 _values <6 μg/ml as compared to other extracts. Therefore further study was confined only to these potent extracts.

**Table 1:** *n vitro *anti-plasmodial activity of plant extracts against K1 strain of *P. falciparum *isolate

**Sl. No**.	Plant extract	**Anti-plasmodial activity (IC**_**50 **_**μg/ml)**		**Cytotoxity IC**_**50 **_**(μg/ml)**	Selectivity Indexes	
				
		A	B		A	B
1.	HA-1	25	22	51.2	2	2.3

2.	HA-2	5.9	5.5	88.9	3.3	16

3.	HA-3	28.6	22.9	19.52	3.1	3.9

4.	HA-4	>30	>15	>90	ND	ND

5.	VC-1	4.1	2.76	12.39	3	4.5

6.	VC-2	23.6	20.9	42.2	1.7	2

7.	VC-3	15.9	13.5	19.52	1.2	1.4

8.	VC-4	>30	>15	>90	ND	ND

Data were shown from three independent experiments

Here, A: Schizont maturation Method [16]; B: pLDH method [17]; Cytotoxicity IC_50 _> 16 μg/ml; non cytotoxic

### Cytotoxic activities and Selective indexes of the plant extracts

Cytotoxic activities of plant extracts are presented in Table 1. The compound was classified as non cytotoxic, when IC_50 _was greater than16 μg/ml [4]. Results revealed that all extracts were non cytotoxic except VC-1 with its IC_50 _Value 12.39 μg/ml. The selective index (SI) were also determined which was related to *in vitro *anti-plasmodial activity [SI = Cytotoxicity (IC_50_)/*in vitro *anti-plasmodial activity (IC_50_)]. VC-1 and HA-2, exhibited 4.5 to 16 fold higher activities against *P. falciparum *than against the rat L-6 cell line respectively (Table 1).

### *In vivo *anti-malarial actions of the plant extracts against *P. berghei*

The anti-malarial activity of the plant extract coded as HA-2 and VC-1 against *P. berghei *infected Swiss albino mice adapting the method of 4-day suppressive test [18] is given in Table 2. After four days of consecutive treatment with the dose of HA-2 extract (15-30 mg/kg), mean parasitaemia (%) in the *P. berghei*-infected mice ranged from 1.5 ± 0.4 to 3.3 ± 1.1 whereas the mean parasitaemia in the control group was 5.1 ± 2.6 (Table 2). Of the different dose ranges, HA-2 extract showed significant inhibition of parasitaemia compared to that of control [overall analysis of variance significance, *F *= 6.9 and *P *= 0.0021 by Scheffe's procedure at α = 0.05]. Mean parasitaemia (%) in the VC-1 extract on dose 10-20 mg/kg ranged from 2.5 ± 1.5 to 5.1 ± 2.0. In the control group the mean parasitaemia was 7.3 ± 4.1 (Table 2). All the dose ranges of VC-1 extract showed significant percent inhibition when compared to control [overall analysis of variance significance, *F *= 4.2 and *P *= 0.017 by Scheffe's procedure at α = 0.05]. Chloroquine (5 mg/kg) was taken as positive control for both extracts.

**Table 2:** *In vivo *anti-malarial activity of plant extracts (HA-2 and VC-1) against *P. berghei *infected Swiss albino mice

**Sl No**.	Plant Extract	Dose	Mean ± SD parasitaemia (%)	Percent suppression of parasitaemia	**ED**_**50 **_**(mg/kg)**	% Survival of animal on day 10
	DMSO	0.1 ml/10 gm B.Wt.	5.1 ± 2.6	-	-	0
	
		10 mg/kg	3.4 ± 1.1	35.2		66
				
1.	HA-2	20 mg/kg	2.0 ± 0.8	63.2	18.29	83
				
		30 mg/kg	1.5 ± 0.4	70.2		100
	
	Chloroquine	5 mg/kg	ND	100	-	100

	DMSO	0.1 ml/10 gm B.Wt.	7.3 ± 4.09	-	-	0
	
		10	5.1 ± 1.9	30.1		50
				
2.	VC-1	15	3.7 ± 0.81	49.3	15.8	66
				
		20	2.5 ± 1.52	63		83
	
	Chloroquine	5 mg/kg	ND	100	-	100

## Discussion

Recently interest has been observed in the screening of plant derived extracts for their anti-malarial activity in order to develop herbal drug against malaria related to traditional medicine. Okokon and Nwafor [22] have demonstrated 75% reduction on 54 mg/kg dose in parasitaemia in mice treated with chloroform extract of *Croton zambesicus *which confirms the folkloric use of this plant in towns of Nigeria.

The plants used in our study were collected from different places in the Garhwal region, where they are being used by the local inhabitants to prepare herbal medicaments. As the Garhwal region covers a wide range of herbs and shrubs approximately 18,440 species of plants and some of them showed good *in vivo *anti-plasmodial activity [23]. The present investigation demonstrates the anti-plasmodial activities of petroleum ether and chloroform extracts of two plants from this region in *P. berghei *infected mice. Treatment with the chloroform *H. antidysenterica *bark extract significantly inhibited parasitaemia compared to no treatment. Simonsen and collaborators [24] found that ethanolic bark crude extract of *H. antidysenterica *had *in vitro *anti-plasmodial activity against chloroquine susceptible strain of *P. falciparum *with its IC_50 _value of 28 μg/ml, while in present study chloroform extract of *H. antidysenterica *was found active against *P. falciparum *isolates (Table 2).

These results are comparable to extracts of some plants [22,25]. On the basis of more reproducible inhibition with chloroform extract of *H. antidysenterica*, assessed for its *in vivo *anti-malarial activity along with cytotoxicity. Percentage suppression of parasitaemia in mice treated with HA-2 extracts showed 70.2% at 30 mg/kg dose (Table 2) with the ED_50 _value of 18.29 mg/kg, indicative of anti-plasmodial potential. Steroidal alkaloids of the conaine and aminopregnane types and the principal one being conessine from the stem bark of *H. antidysenterica *was reported previously [26]. Zirihi and co-workers [27] evaluated *in vitro *anti-plasmodial activity of conessine from the plant *Funtumia elastica *against chloroquine-resistant strain FcB1 of *P. falciparum *and cytotoxity against a rat cell line L-6 with its IC_50 _1.04 μg/ml and 14 μg/ml respectively. The presence of conessine as leading compound may be responsible for the anti-malarial activity of extract HA-2.

In case of *V. canescens *treatment with the petroleum ether extract inhibits parasitaemia significantly compared to that of control. However *V. canescens *which is used against malaria in the Garhwal region has not been investigated till now. The petroleum ether extract of *V. canescens *whole plant extract showed slightly cytotoxic effect against L-6 cells of rat skeletal myoblast (IC_50 _12.39 μg/ml) and the inhibition of parasite growth of *P. falciparum *(IC_50 _2.76 μg/ml). Similarly petroleum ether extracts of *Viola websteri *Hemsl (Violaceae) were reported to have anti-plasmodial activity against *P. falciparum in-vitro *[28]. VC-1 is evaluated for its *in vivo *anti-malarial activity in *P. berghei *infected Swiss albino mice as it showed significant anti-plasmodial activity against *P. falciparum *isolate. The percent reduction of parasitaemia of VC-1 extract was found 63% at 20 mg/kg dose with the ED_50 _value of 15.8 mg/kg against *P. berghei *infected mice (Table 2). Results from *in vivo *anti-malarial activity revealed that VC-1 showed significant reduction of parasitaemia on dose ranges 10-20 mg/kg. Where as on administration of the dose of 30 mg/kg of VC-1, weight loss was recorded in some mice and 30% of the mice were died with in 10 days.

## Conclusion

The present investigation demonstrates the anti-malarial effects of chloroform and petroleum ether extracts from two commonly used medicinal plants in the Garhwal region. Efforts will be undertaken to continue the biologically guided fractions in order to isolate and identify the active ingredient as well as to understand the mechanism of inhibition. Since herbal products are globally used and the bio-active compounds isolated from these plants may work as bio-marker in variety of herbal formulations. These formulations may meet out the need of time against malaria.

## Competing interests

The authors declare that they have no competing interests.

## Authors' contributions

GV (PhD student) contributed in the laboratory work, analysis of the data and drafted the paper. VKD and DDA designed the work and supervised the *in vitro *and *in vivo *studies and analysed the data. PKA performed the *in vivo *studies. All authors contributed to the critical review of the manuscript and agree to submission.
